# Additively Manufactured Maraging Steel: Influence of Heat Treatment on Corrosion and Mechanical Properties

**DOI:** 10.3390/ma18091999

**Published:** 2025-04-28

**Authors:** Daniel Pustički, Željko Alar, Zvonimir Bandov

**Affiliations:** Faculty of Mechanical Engineering and Naval Architecture, University of Zagreb, Ivana Lučića 5, 10000 Zagreb, Croatia; zeljko.alar@fsb.unizg.hr (Ž.A.);

**Keywords:** additive manufacturing, LPBF, maraging steel, microstructure, corrosion, hardness

## Abstract

The advancement of additive manufacturing (AM) technologies, particularly laser powder bed fusion (LPBF), has enabled the production of complex components with enhanced mechanical properties and shorter lead times compared to conventional manufacturing processes. This study focuses on the characterization of maraging steel (EOS MS1) fabricated by LPBF technology using an EOS M 290 system. Three material groups were investigated: a conventionally manufactured tool steel (95MnWCr5) serving as a reference, LPBF-produced maraging steel in the as-built condition, and LPBF-produced maraging steel subjected to post-processing heat treatment. The samples were thoroughly examined using optical microscopy, scanning electron microscopy (SEM), energy-dispersive X-ray spectroscopy (EDS), glow discharge optical emission spectroscopy (GDOES), electrochemical corrosion analyses in a 3.5% NaCl solution, and Vickers microhardness measurements. Electrochemical tests revealed that heat-treated LPBF maraging steel samples exhibited slightly increased corrosion current densities relative to their as-built counterparts, attributed to the formation of Ti-rich and Ni-rich precipitates during aging, creating localized microgalvanic cells. Despite the increased corrosion susceptibility, hardness measurements clearly demonstrated enhanced hardness and mechanical properties in heat-treated samples compared to the as-built state and conventional tool steel reference. The findings underscore the importance of optimized LPBF parameters and controlled post-processing heat treatments in balancing mechanical performance and corrosion resistance. Consequently, LPBF-produced maraging steels hold considerable promise for tooling and industrial applications where high strength, dimensional stability, and acceptable corrosion behavior are required.

## 1. Introduction

**Figure 1:**
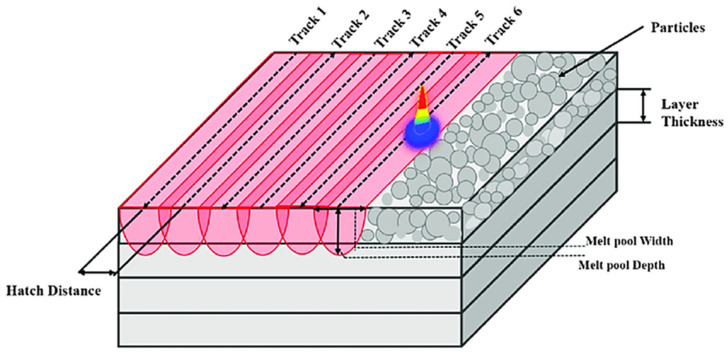
LPBF process parameters [6].

Additive manufacturing has revolutionized the fabrication of complex metal parts by enabling layer-by-layer consolidation without many of the geometrical constraints inherent to traditional processes [1]. Recent work underscores the advantages of AM, particularly the laser powder bed fusion (LPBF) technique, in fields ranging from aerospace to biomedical engineering, largely due to the process’s ability to produce highly dense and complex geometry components [2,3]. Before the LPBF procedure, it is crucial to optimize the relevant parameters (Figure 1) to ensure successful part fabrication and avoid microcracks or part failure. Through effective parameter optimization, the molten layer fuses properly with the previously solidified layer, leading to satisfactory relative density. Improved resolution and powder flowability can be achieved by adjusting the particle size distribution [1,4,5].

Process parameters are directly linked to porosity and layer morphology imperfections. Inadequate parameter optimization may cause the excessive overlap of laser scans and thus introduce defects that compromise the model’s homogeneity and increase porosity [7]. To achieve high density and refine layer morphology, methods such as re-melting and varied scanning strategies are applied to reduce residual stress [8].

A significant part of the research on LPBF focuses on titanium, nickel, and iron powders [6,7,8,9]. As LPBF technologies evolve, other metals, like copper, tungsten, magnesium, and aluminum, are increasingly considered [9,10,11,12,13]. Investigations on various tool and stainless steels indicate that using higher laser power (*P*,W) and a constant scanning speed stabilizes the melting flow, facilitating the fabrication of fully dense components [14].

Maraging steels offer a low carbon content, high nickel and cobalt contents, and excellent mechanical properties after aging heat treatment [15]. Maraging steels are frequently used in applications exposed to corrosive environments, including tooling and aerospace components, where optimal corrosion resistance alongside high mechanical performance is critically important. Prior research has demonstrated that, when properly optimized, maraging steel exhibits superior strength, toughness, and dimensional stability, which are properties that are crucial in the production of referent materials, especially hardness reference blocks [14,15]. Nevertheless, achieving consistent quality can be challenging because of potential defects such as a lack of fusion (LOF), internal porosity, and residual surface oxides. 

Multiple studies have shown that these defects can represent stress concentrators and sites for corrosion initiation in aggressive media [16,17]. Consequently, the correlation between microstructural defects and the electrochemical behavior of LPBF-fabricated steel has been a subject of active investigation. Previous research highlights the importance of laser power, scanning speed, and layer thickness; optimizing these parameters can reduce porosity, minimize microcracking, and enhance corrosion resistance [18]. In parallel, the significance of hardness reference blocks has been noted, as corrosion phenomena can alter the calibration surfaces of such blocks, ultimately compromising measurement integrity. Maintaining consistent hardness standards is vital in industrial contexts, where the dimensional stability and calibration accuracy of such blocks must be protected from any degradation due to corrosive environments.

Several investigations have shown that LPBF maraging steels benefit from post-build heat treatments, which help homogenize the microstructure, reduce internal stresses, and potentially improve both mechanical strength and corrosion resistance [15,16,17,18,19]. Meanwhile, direct comparisons with conventionally produced tool steels, like 95MnWCr5, indicate that maraging steel fabricated by LPBF can attain equivalent or superior performance under certain conditions [20]. However, achieving high corrosion resistance also demands controlling oxygen and humidity levels in the build chamber, as well as ensuring minimal post-build oxidation. Furthermore, the addition of hardness testing complements typical mechanical and electrochemical evaluations by allowing for the direct assessment of local property variations, especially near or within defect-rich regions [19,21].

In this study, maraging steel specimens produced by LPBF were analyzed to evaluate the impact of manufacturing conditions and subsequent heat treatment on microstructural features and to connect hardness and corrosion behavior. This study specifically investigates maraging steels produced by the LPBF method, while conventional tool steel (95MnWCr5) is used in the form of a hardness reference block. It serves as a reference due to its well-defined chemical composition, high microstructural homogeneity, and excellent mechanical stability, making the specifics of its original manufacturing route irrelevant for the purpose of this comparative analysis. The aim is to establish an optimal balance between improved mechanical performance and acceptable corrosion resistance for application in reference hardness blocks.

## 2. Materials and Methods

All samples were fabricated using an EOS M290 LPBF system (EOS GmbH, Munich, Germany) under an inert argon atmosphere to prevent oxidation. The chosen material was EOS MS1 maraging steel powder, classified as 18Ni-300 maraging steel, with a nominal chemical composition (wt. %) of Ni (17–19%), Co (8.5–10%), Mo (4.5–5.2%), Ti (0.5–1.2%), Si (0.02–0.05%), Cr (0.24–0.32%), Mn (0.05%), P (0.006–0.014%), and S (0.002–0.003%), with a carbon content below 0.03% [22]. 

**Figure 2:**
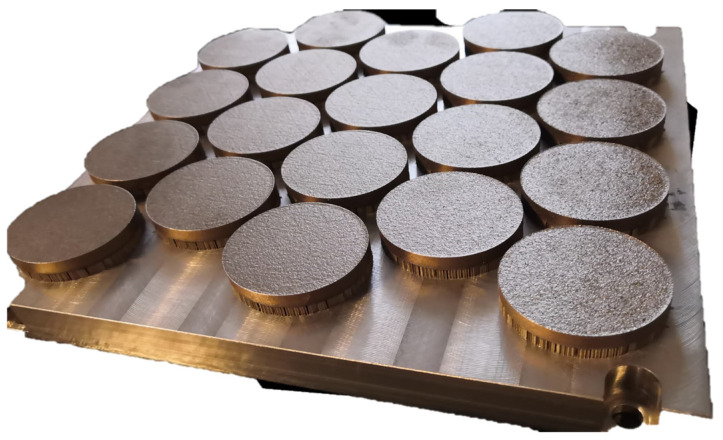
Samples after the LPBF process on the base plate.

An optimal volumetric energy density (LED) of 55 J/mm^3^ was obtained by adjusting the following parameters: laser power at 305 W, scan speed at 1010 mm/s, hatch spacing of 0.11 mm, and a layer thickness of 50 µm. The LED used for sample fabrication was established through preliminary experimental trials and validated by comparison with relevant literature data, ensuring the near-full densification and minimal residual porosity in the produced LPBF specimens [15,23,24,25]. After the powder was deposited and each layer laser exposed, the base plate was incrementally lowered by 50 µm until a final cylindrical geometry (*Ø* = 50 mm, *h* = 10 mm) was achieved, as shown in Figure 2. Three distinct types of samples were used, with U1 as a reference sample with the same dimensions as the fabricated samples, and five identical specimens of samples U2 and U3:

- U1: Conventionally manufactured 95MnWCr5 tool steel for use as a hardness reference block.
- U2: Maraging steel samples produced by LPBF in the as-built condition.
- U3: Maraging steel samples produced by LPBF and subsequently subjected to a post-build heat treatment.

After fabrication via LPBF, samples were carefully removed from the base plate using a band saw, milled to remove supports, and then prepared through standard metallographic techniques involving grinding and polishing. The U3 samples underwent post-build heat treatment (aging) at 490 °C for 4.5 h and were cooled in air to stabilize the martensitic structure and activate the precipitation-hardening process. All specimens were subsequently ground, polished, and cleaned in an ultrasonic bath before characterization and hardness measurement. Density measurements, essential for evaluating porosity, were performed by hydrostatic weighing in pentadecane. The theoretical density of maraging steel (8100 kg/m^3^) served as a reference for calculating relative density.

Elemental composition was verified using GDOES, particularly to confirm the presence and concentration of alloying elements (Ni, Co, Ti, Cr) and to detect minor oxidation or contamination. Microstructural features were characterized via an optical OLYMPUS GX51F-5 light microscope with a DP-25 CCD camera (Olympus Corporation, Shinjuku City, Tokyo, Japan). SEM analysis was carried out on a TESCAN VEGA TS5136LS device with EDS (TESCAN, Brno, Czech Republic).

A hardness test was introduced to complement the microstructural and electrochemical investigations. A ZwickRoell ZHVμ-ST micro-Vickers hardness tester (Indentec Ltd., West Midlands, UK) was used on the polished flat surface of the samples. Ten randomly selected points on each specimen’s polished face were tested to capture the surface and cross-section variability of hardness. Hardness measurements were conducted under a load of 4.903 N, with a loading speed of 0.1 mm/s and a dwell time of 15 s. Microhardness and elastic modulus measurements were also conducted using an Anton Paar STEP 100 microindentation device (Anton Paar GmbH, Graz, Austria). The instrument operates under a controlled indentation protocol of loading and unloading to 5000 mN at a rate of 266.67 mN/s and holding time at maximum load of 15 s. Each indentation was performed at a load of 5 N, allowing for both hardness (Vickers scale) and elastic modulus determination from the load–displacement curve in accordance with the Oliver–Pharr method.

Electrochemical tests were performed on a potenciostat/galvanostat (Princeton Applied Researche-Amatek Versa STAT, Berwyn, PA, USA) device. Corrosion performance was evaluated in a 3.5 % NaCl solution at room temperature using a three-electrode setup: a saturated calomel electrode as a reference, platinum mesh as the counter electrode, and the investigated samples as working electrodes. The exposed surface area in the electrochemical cell was 1 cm^2^. Linear polarization resistance (LPR) was applied in a ±20 mV range around the open-circuit potential at 0.166 mV/s to measure the polarization resistance, *R*_p_, kΩ. The Tafel extrapolation was performed over ±250 mV from the corrosion potential to determine the corrosion current density (*j*_cor_) and corrosion rate (*v*_cor_) of all 3 sample types.

## 3. Results

### 3.1. Chemical Composition and Density 

Table 1 summarizes the chemical composition of the three groups of samples. The maraging steel samples (U2 and U3) match the expected low carbon and high Ni, Co, and Mo contents. The slight oxidation suggests minimal surface contamination, which might be further minimized by improved chamber sealing or storage protocols. By contrast, the conventional tool steel (U1) showed higher carbon and chromium content, characteristic of its composition for hardness reference blocks.

The sample U1, being produced by a traditional manufacturing process, was considered to exhibit 100% of its theoretical density. In comparison, LPBF-produced maraging steel samples U2 and U3 achieved relative densities of 99.36 % (8049.4 kg/m^3^) and 99.46 % (8057.2 kg/m^3^), respectively, confirming the high densification and low porosity levels attainable through optimized LPBF parameters.

### 3.2. Microstructural Observations

**Figure 3:**
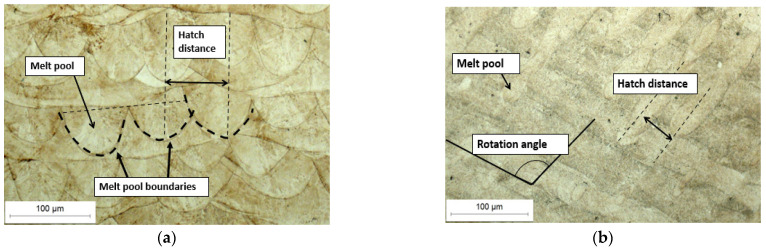
Microstructure of specimen U2, etched: (**a**) cross-section, (**b**) longitudinal section.

**Figure 4:**
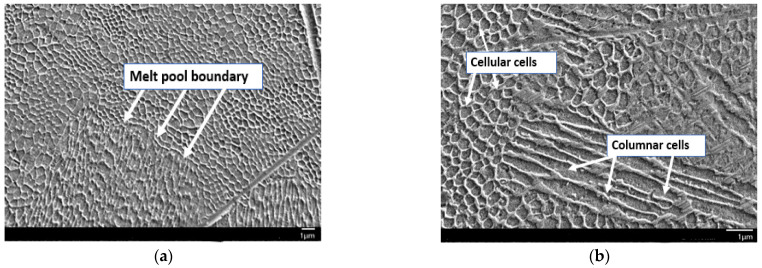
SEM: (**a**) melt pool boundary, (**b**) cellular–dendritic microstructure in sample U2.

The analysis was carried out on both the transversal and longitudinal sections of the manufactured samples. Optical and SEM examinations of the etched samples revealed a structure characteristic of the LPBF process. Figure 3a shows the cross-section of the U1 specimen in its as-built state, where the laser scan paths and the melting pool boundaries are clearly visible. Figure 3b then presents the longitudinal section of the same specimen, which similarly reveals the characteristic laser traversal paths. Furthermore, SEM analysis (Figure 4a) confirms the pronounced melting pool boundaries and reveals a columnar cell structure (Figure 4b) at both longitudinal and transverse orientations, depending on the examined cross-section of the sample. The observed cellular–dendritic microstructure was uniform and regular, consistent with typical microstructural characteristics expected in materials fabricated by the LPBF process. No uniform uniaxial growth of the cellular structure was observed, resulting in the absence of a consistently oriented cell pattern [25].

In addition to the standard microstructural characterization of LPBF samples, special attention was paid to the identification and analysis of process-induced defects. Optical and SEM microscopy were employed to examine porosity, lack-of-fusion (LOF) zones, and oxide inclusions, which may critically affect the mechanical integrity and corrosion behavior of LPBF-produced maraging steel. 

**Figure 5:**
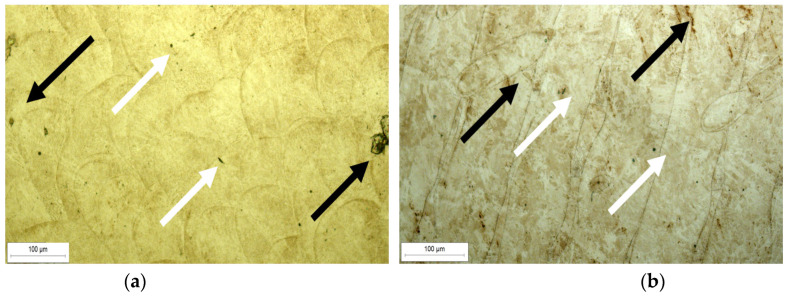
Structural defects in sample U2: (**a**) transverse section pores (white arrows) and inclusions (black arrows), (**b**) longitudinal section pores (white arrows) and inclusions (black arrows).

Figure 5 shows optical micrographs of LPBF-fabricated samples with small, pore-like features and local discontinuities. White arrows indicate larger pore clusters, typically formed due to insufficient laser energy input or suboptimal scan overlap, which results in incomplete melting between adjacent tracks. Black arrows highlight finer internal inclusions and defects, likely originating from trapped gas, incomplete powder fusion, or oxide contamination introduced during powder handling or within the EOS M290 chamber.

**Figure 6:**
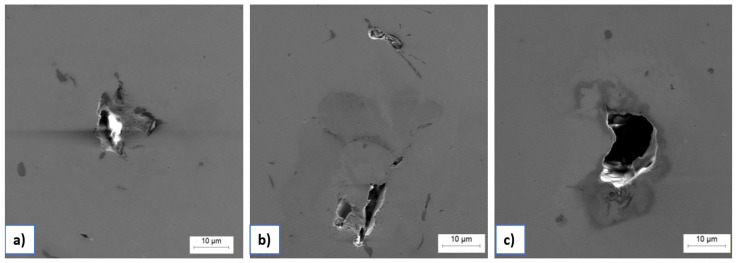
Structural defects under SEM: (**a**) LOF, (**b**) LOF + pores, (**c**) pore.

Further SEM analysis of unetched LPBF manufactured samples (Figure 6) revealed some inter-layer defects such as LOF, irregular inclusions, and micro-porosity. These features often appear along melt pool boundaries and layer interfaces, acting as stress concentrators and electrochemically active zones. Their presence is particularly relevant in corrosive environments, where they can promote localized corrosion mechanisms such as pitting or microgalvanic corrosion.

**Figure 7:**
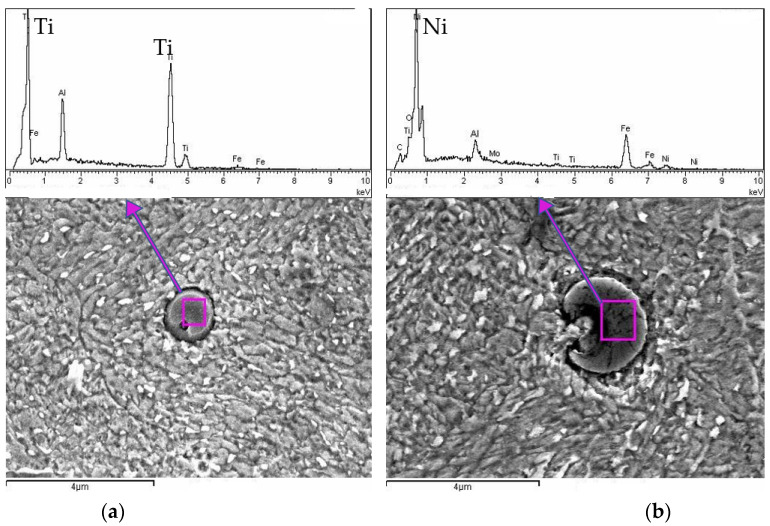
SEM EDX analysis of sample U3: (**a**) Ti precipitates, (**b**) Ni precipitates.

To further investigate the microstructure of the heat-treated sample and validate observations from the SEM analysis, an additional detailed analysis was performed using scanning electron microscopy coupled with EDS. The goal of this step was to precisely identify and verify the chemical composition of the precipitates previously noted in the SEM observations. The results, displayed in Figure 7, clearly reveal spherical precipitates embedded within the steel matrix.

**Figure 8:**
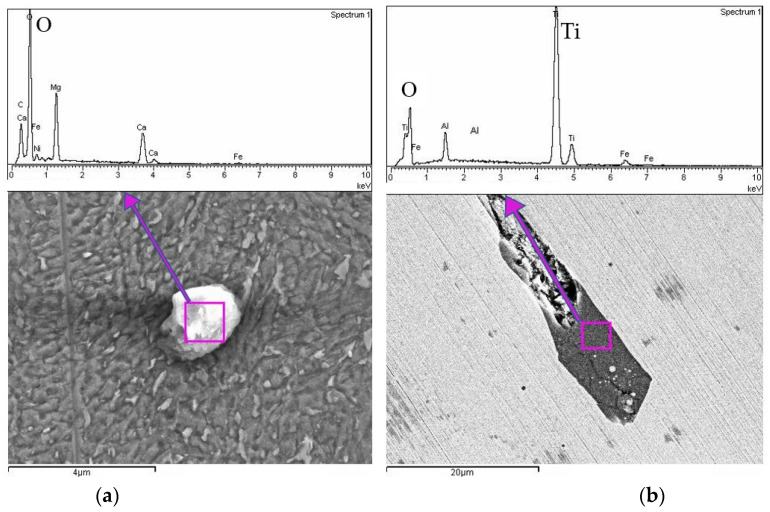
SEM EDX analysis of sample U3: (**a**) oxide inclusion, (**b**) Ti-oxide inclusion on a polished sample.

Both SEM images display distinctive spherical inclusions, surrounded by a clear interface separating them from the matrix. The EDS spectra reveal prominent peaks corresponding to Ti (Figure 7a) and Ni (Figure 7b), suggesting that these spherical inclusions are primarily metallic Ti- and Ni-based precipitates. Other minor elemental peaks (such as Al and Si) also appear, indicating possible alloying additions or contamination within or adjacent to the precipitates. The Fe peaks dominate the spectra outside the precipitates, as expected for a steel-based matrix. In addition to the metallic precipitates identified in Figure 7, further SEM-EDS analysis of sample U3 revealed the presence of distinct oxide inclusions, shown in Figure 8. 

These inclusions were characterized at different magnifications and exhibit varied chemical compositions and morphologies. The spherical inclusion in Figure 8a is rich in oxygen, magnesium, and calcium, suggesting the formation of a complex oxide particle. In contrast, the elongated feature in Figure 8b exhibits strong titanium and oxygen peaks, indicating the presence of Ti oxides embedded within the matrix. Their detection aligns with prior reports that highlight the tendency of maraging steels to develop oxide layers or particles, particularly when exposed to air during processing or post-treatment. These inclusions may locally alter electrochemical behavior, potentially acting as preferential corrosion initiation sites.

### 3.3. Hardness Measurement

The results of hardness measurements obtained from ten indentations (*n* = 10 per group) and performed using the HV0.5 method is summarized in Table 2.

The conventional tool steel showed hardness values consistent with the typical properties of high-carbon tool steels commonly used as calibration blocks. For the LPBF-built MS1 in the as-printed condition, local hardness variations were observed, likely associated with microscale defects such as porosity and microcracks. In the case of the LPBF-built, post-treated MS1 samples, an increase in hardness values was evident following the aging process, reflecting the precipitation-hardening effect characteristic of maraging steels. 

In addition to standard HV0.5 hardness testing, ten microindentations per sample were performed (*n* = 10) under a 5 N load to assess both the Vickers hardness and the elastic modulus of the material. The results are summarized in Table 3. 

### 3.4. Electrochemical Behavior

**Figure 9:**
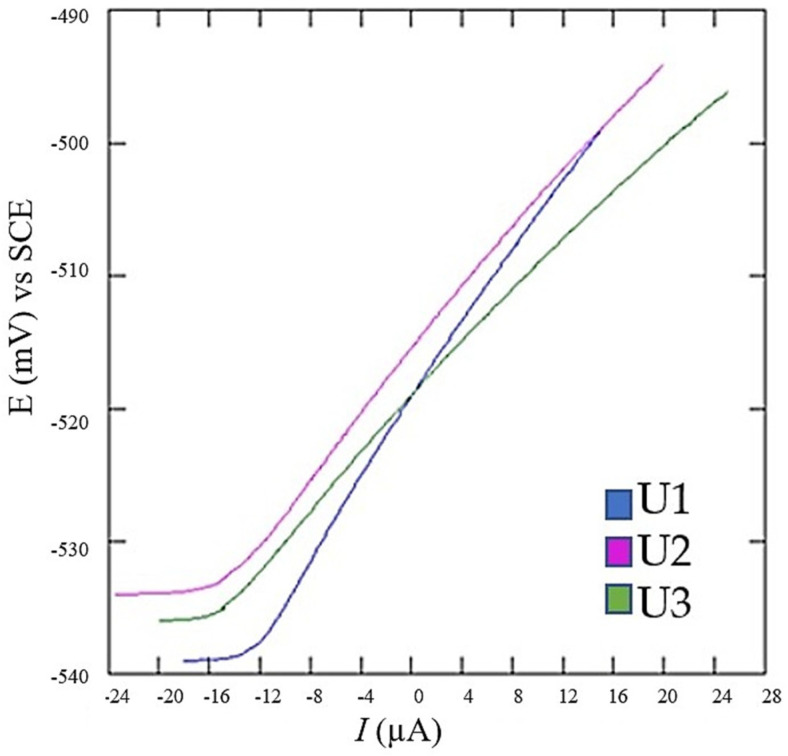
Linear polarization curves.

Linear polarization measurements were carried out at a potential range of ±20 mV relative to the corrosion potential, with a polarization rate of 0.166 mV/s, using the VersaStudio software, V 2.262 (Amatek Versa STAT, Berwyn, PA, USA). The resulting linear polarization curves are presented in Figure 9, while Table 4 summarizes the corresponding mean values and standard deviation of corrosion potential (*E*), corrosion current density (*I*_cor_), corrosion rate (*v_c_*_or_), and polarization resistance (*R*_p_) after five measurements per group.

**Figure 10:**
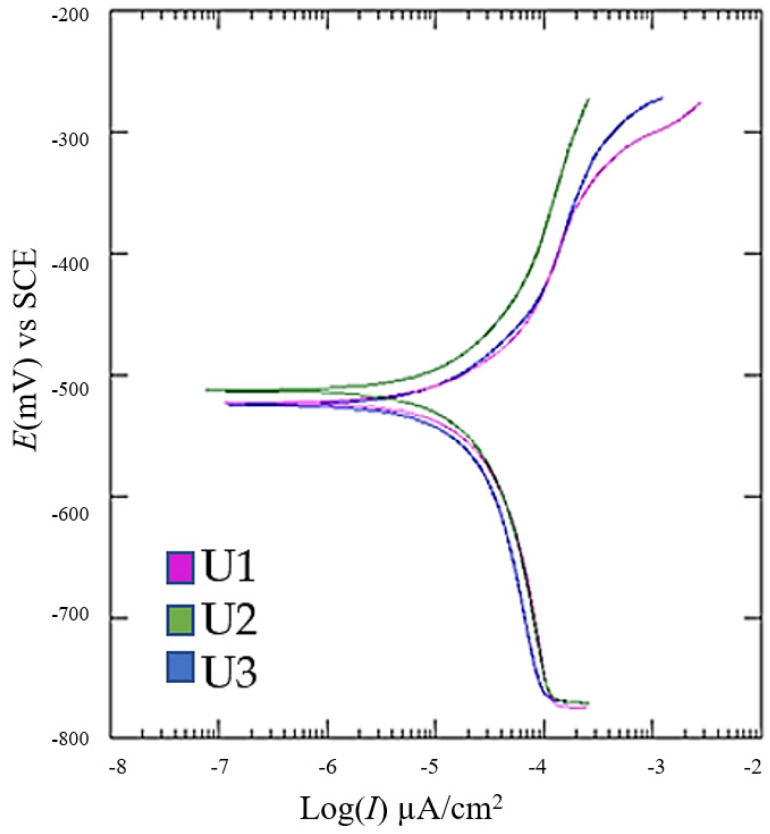
Potentiodynamic polarization curves.

Quasi-potentiodynamic polarization was conducted within a potential range of ±250 mV from the corrosion potential at a polarization rate of 0.1666 mV/s to record the polarization curves. Using the Tafel extrapolation method, the corrosion potential *E* (*I* = 0), corrosion current density *j*_cor_, and corrosion rate *v*_cor_, as well as the anodic *β*_A_ and cathodic *β*_K_ slopes, were determined after five measurements per group. The mean values and standard deviations of the parameters are presented in Table 5, while the corresponding Tafel extrapolation diagrams are shown in Figure 10.

The Tafel extrapolation shows that the heat-treated samples (U3) had the highest corrosion current density and thus a marginally elevated corrosion rate, whereas the as-build samples (U2) performed slightly better under the test conditions. These differences likely stem from microstructural changes during heat treatment and the potential formation of surface oxides or microcracks exacerbated by thermal cycling.

## 4. Discussion

The findings of this study highlight the complex correlation between the chemical composition, microstructural integrity, and functional performance of LPBF-produced maraging steel under different processing conditions. As expected, both U2 and U3 samples closely match the nominal composition of EOS MS1 powder, with high Ni, Co, and Mo content and low levels of C, P, and S, ensuring good precipitation potential. In contrast, the U1 sample exhibited significantly higher carbon and chromium content, consistent with its classification as a high-carbon tool steel, optimized for dimensional stability and wear resistance. Density measurements confirmed that LPBF processing yielded highly consolidated structures, with relative densities of 99.36% for U2 and 99.46% for U3. These values are in excellent agreement with prior studies on maraging steel processed by LPBF and support the claim that optimized process parameters can produce nearly fully dense components [15,25,26,27,28].

Detailed SEM-EDS analysis performed on the samples revealed spherical intermetal phases in the U3 sample, distinctly separated from the surrounding Fe-based matrix. The heat treatment of maraging steels significantly increases hardness through precipitation hardening, primarily by forming fine, uniformly distributed Ti-rich and Ni-rich intermetallic precipitates within the martensitic matrix [28]. Despite this beneficial mechanical enhancement, heat treatment also introduces localized compositional gradients and intermetallic phases. These precipitates act as microgalvanic cells, leading to local differences in electrochemical potential and, consequently, increased susceptibility to corrosion [29,30]. Although the overall densification level of the LPBF samples was high (>99.3%), even small defects, especially those connected to the surface, can compromise corrosion resistance. This is reflected in the higher corrosion current densities measured for heat-treated samples, where thermal cycles may have enhanced oxide formation at defect sites.

These results emphasize the importance of not only achieving high global density but also minimizing process-induced defects to ensure stable corrosion behavior in service conditions.

SEM-EDS analysis clearly identified these metallic Ti-rich and Ni-rich precipitates, confirming their formation during solidification or subsequent aging processes and establishing their critical role as initiation sites for localized corrosion. Additional SEM-EDS analysis revealed oxide inclusions within the maraging steel matrix, including spherical and elongated features rich in O, Mg, and Ti. These oxides likely originate from the LPBF process or powder atomization, especially in areas with suboptimal shielding or surface exposure. Their presence may contribute to localized corrosion by introducing chemical heterogeneity, reinforcing the need for strict atmospheric control and potential surface finishing in critical applications.

The experimental data from hardness tests clearly reflect the influence of microstructure on mechanical properties. The conventionally manufactured tool steel reference sample exhibited expected hardness values (575 ± 8.3 HV0.5 cross-section, 572 ± 6.3 HV0.5 surface), consistent with its high carbon content and refined microstructure. These findings were corroborated by instrumented indentation testing under a 5 N load, which yielded a similar average hardness of 588 ± 5.9 HV.

In contrast, the as-built LPBF maraging steel sample U2 demonstrated hardness levels typical for maraging steels in the non-aged state (355 ± 4.5 HV0.5 cross-section, 358 ± 4.1 HV0.5 surface), and similar values were obtained from indentation testing (360 ± 6.5 HV). These reduced values are attributed to residual porosity, lack-of-fusion defects, and the absence of hardening precipitates in the as-built condition.

The heat-treated LPBF maraging steel sample U3 achieved the highest hardness values among all samples (598 ± 5.1 HV0.5 cross-section, 597 ± 4.9 HV0.5 surface), clearly confirming the beneficial effect of precipitation hardening. This is further supported by the indentation method, which showed a hardness of 601 ± 6.2 HV, reflecting a more homogeneous and stiffened microstructure due to the formation of Ni- and Ti-based precipitates. Notably, the lower standard deviation in hardness for U3 compared to U2 also indicates improved microstructural uniformity after heat treatment. 

The conventional tool steel sample U1 demonstrated a relatively high elastic modulus (239.2 ± 5.3 GPa), indicative of its dense and homogeneous microstructure typical of forged tool steels. In comparison, the sample U3 exhibited a slightly higher modulus (245.1 ± 10.1 GPa), which can be attributed to precipitation hardening and increased microstructural stiffness. Conversely, the as-built LPBF sample U2 showed a significantly lower modulus (191.3 ± 11.5 GPa) due to the absence of homogenization and hardening precipitates, highlighting the effect of post-processing on restoring the mechanical integrity of additively manufactured components.

However, electrochemical measurements exhibited an inverse correlation between hardness and corrosion resistance. Heat-treated LPBF samples showed the highest corrosion rates, as confirmed by linear polarization (*j*_cor_ = 21.6 µA, *R*_p_ = 1.003 kΩ) and Tafel extrapolation methods (*j_cor_* = 31.98 µA). Although initially counterintuitive, this phenomenon is explained by localized microgalvanic corrosion induced by precipitates formed during aging. Conversely, the conventional tool steel reference (U1) achieved better corrosion resistance (*R*_p_ = 1.462 kΩ, *j_cor_* = 14.9 µA), indicating fewer microstructural heterogeneities and, consequently, reduced microgalvanic effects.

## 5. Conclusions

Based on the experimental results obtained, the following conclusions can be made:

- Conventional 95MnWCr5 reference blocks exhibited the highest hardness among all materials tested, while the heat-treated maraging steel showed a clear hardness improvement compared to its as-built counterpart.
- Detailed SEM-EDS analyses confirmed the presence of precipitates, clearly linking their elemental composition and distinct morphology to the observed corrosion behavior. Furthermore, sub-surface pores and oxides in LPBF maraging steels can significantly reduce corrosion resistance despite high overall density.
- Electrochemical corrosion tests performed in a 3.5% NaCl solution indicated acceptable corrosion resistance for both as-built and heat-treated LPBF maraging steels, although the heat-treated samples showed increased corrosion current densities. This somewhat counterintuitive phenomenon is attributed to the formation of Ti-rich and Ni-rich precipitates during aging, which, despite enhancing hardness and mechanical properties, create localized electrochemical heterogeneities and microgalvanic cells, thus increasing susceptibility to corrosion.
- Consequently, maraging steels fabricated by LPBF present significant promise for industrial applications requiring superior hardness and dimensional stability, provided that careful attention is paid to post-treatment processes and the balance between mechanical enhancement and corrosion susceptibility. Furthermore, if these materials are intended for use as hardness reference blocks or in aggressively corrosive environments, additional protective coatings or measures are recommended to maintain calibration reliability and component integrity.

Future work may include a broader range of LPBF parameters and heat treatment temperatures, in-depth residual stress measurements, and extended immersion testing to elucidate the long-term corrosion and mechanical stability of LPBF maraging steels in various service conditions. Variations in oxide layer thickness occurring at elevated temperatures can significantly influence the corrosion resistance and mechanical durability of maraging steels; thus, further studies will consider this factor during heat treatment optimization.

## Figures and Tables

**Table 1:** Chemical composition of samples (wt. %).

wt. %	C	Si	Mn	P	S	Cr	Mo	Ni	Co	Ti	Al	Fe
U1	0.9	00.4	1.05	0.03	0.03	0.65	-	-	-	-	-	Bal.
U2	0.02	0.05	0.10	0.006	0.003	0.32	4.22	16.68	9.7	0.71	0.12	Bal.
U3	0.02	0.05	0.10	0.006	0.003	0.32	4.22	16.68	9.7	0.71	0.12	Bal.

**Table 2:** Mean values and standard deviations of HV0.5 measurements.

Sample	HV0.5	HV0.5
	Cross-section	Surface
U1	575 ± 8.3	572 ± 6.3
U2	355 ± 4.5	358 ± 4.1
U3	598 ± 5.1	597 ± 4.9

**Table 3:** Mean values and standard deviations of instrumented indentation testing.

Sample	Load [N]	Young’s Modulus [GPa]	Hardness [HV]
U1	5	239.2 ± 5.3	588 ± 5.9
U2	5	191.3 ± 11.5	360 ± 6.5
U3	5	245.1 ± 10.1	601 ± 6.2

**Table 4:** Electrochemical parameter mean values obtained from linear polarization curves.

Sample	*E* (*I* = 0), mV	*j*_cor_, µA	*v*_cor_, mm/god	*R*_p_, kΩ
U1	−589.4 ± 1.61	14.9 ± 0.80	0.175 ± 0.01	1.462 ± 0.22
U2	−515.7 ± 1.22	18.2 ± 0.65	0.214 ± 0.02	1.190 ± 0.12
U3	−519.4 ± 2.33	21.6 ± 1.05	0.254 ± 0.02	1.003 ± 0.42

**Table 5:** Mean electrochemical parameters obtained from potentiodynamic polarization diagrams.

Sample	*E*(*I =* 0), mV	*j*_cor_, µA	*v*_cor_, mm/god	*β*_A_*,* V/dek	*β*_K_*,* V/dek
U1	−513.3 ± 1.52	28.90 ± 0.60	0.339 ± 0.04	0.277 ± 0.18	0.433 ± 0.20
U2	−525.7 ± 1.15	25.26 ± 0.55	0.297 ± 0.03	0.186 ± 0.10	0.419 ± 0.15
U3	−523.7 ± 2.55	31.98 ± 1.50	0.375 ± 0.05	0.172 ± 0.15	0.443 ± 0.20

## Data Availability

The data presented in this study are an extension of a final thesis openly available in [Repository of Faculty of Mechanical Engineering and Naval Architecture University of Zagreb] at https://repozitorij.fsb.unizg.hr/islandora/object/fsb:10445 (accessed on 21 March 2025).
